# Long-Term Stability of the Use of Patient Specific Implants in Orthognathic Surgery: A Systematic Review

**DOI:** 10.1016/j.identj.2026.109503

**Published:** 2026-03-19

**Authors:** Aaron Wu, Hui Wen Tay, Yiu Yan Leung

**Affiliations:** Oral and Maxillofacial Surgery, Faculty of Dentistry, The University of Hong Kong, Hong Kong, China

**Keywords:** Orthognathic surgery, Dentofacial deformity, 3D printing, CAD/CAM, Patient-specific implant, Custom plate, Long-term stability, Relapse

## Abstract

**Introduction and aim:**

This systematic review aimed to evaluate the long-term stability of patient-specific implants (PSIs) in orthognathic surgery.

**Methods:**

The review was conducted based on the Preferred Reporting Items for Systematic Reviews and Meta-Analyses (PRISMA) statement. A systematic search including an electronic search of numerous databases with keywords, manual and reference searches was performed. The search results were evaluated and underwent 2 rounds of elimination, first by title and abstract and second by fulfilling the 6 predetermined criteria. The included studies underwent the final systematic review.

**Results:**

Six studies with a total of 171 subjects were included in the final review. The long-term stability of the use of PSIs in orthognathic surgery ranged from 0.27 mm to 0.5 mm for the x-axis and 0.3 mm to 1.07 mm for the y-axis. The mean change was 0.1°, 0.5° and 0.2° for the pitch, roll and yaw, respectively. The absolute mean difference of the SNA angle was 0.57°. The mean duration of operation for using PSIs was shorter than the use of conventional miniplates in orthognathic surgery by 15.7%. Complications and reoperation rates when using PSIs in orthognathic surgery were reported to be 0 in 3 studies and not reported in the other 3 studies.

**Conclusion:**

The results of this systematic review indicate that PSIs demonstrate clinically acceptable stability over the medium-to-long term and represent a reliable method of fixation in orthognathic surgery. Additional high-quality studies with standardised methodology and longer follow-up period of more than 24 months are recommended.

**Clinical relevance:**

This systematic review highlights the high level of long-term stability when using PSIs in orthognathic surgery in terms of bodily movements, complications and reoperation rates. Oral and maxillofacial surgeons and biomechanical engineers may now have a more comprehensive and deeper understanding of the use of PSIs in the long term, which can assist them in choosing the method of fixation in orthognathic surgery.

## Introduction

Orthognathic surgery is a well-established surgical intervention aimed at correcting jaw deformities, improving facial aesthetics and restoring proper occlusal function. Over the years, the use of fixation devices has played a critical role in ensuring the success of these procedures. Historically, conventional miniplates have been the gold standard for fixation, offering a reliable solution with a long history of clinical success. These devices have proven effective in stabilising bone segments during the healing and remodelling processes, and their widespread use has established a solid foundation for predictable outcomes in orthognathic surgery. Thanks to advancements in technology such as computer-aided design / computer-aided manufacturing (CAD/CAM) and virtual surgical planning (VSP), titanium fabricated patient-specific implants (PSIs) are being adopted by increasingly more oral and maxillofacial surgeons in the field of orthognathic surgeries.

PSIs are custom-designed implants tailored to the unique anatomical structures of each patient, which contributes to their high level of accuracy in positioning and fixation.^1^, ^2^, ^3^ As this technology gains popularity, it has been the subject of growing interest in the clinical and research communities. Numerous reviews and studies have already explored and noted PSIs’ high level of accuracy.^4^^,^^5^ These analyses have highlighted the potential of PSIs to improve surgical outcomes and streamline procedures. Many long-term studies on accuracy of PSIs for orthognathic surgery did not present radiographic evidence.^6^, ^7^, ^8^, ^9^ Hence, despite the increasing adaptation of PSIs in orthognathic surgeries, there remains a significant gap in the literature knowledge regarding their long-term stability with updated radiographies to compare preoperative and long-term postoperative data.

Long-term stability is a critical measure of success in orthognathic surgery because it reflects the ability of fixation methods to maintain the corrected skeletal position over extended periods. Traditional or conventional fixation using miniplates has demonstrated efficacy in this regard,^10^^,^^11^ but the same cannot yet be definitively said of PSIs because there is currently a lack of literature focusing specifically on this outcome. This is particularly noteworthy for follow-up periods of at least 6-12 months with radiographic data, which are essential for assessing the long-term stability of these implants.

The specific aim of this systematic review was to address this literature knowledge gap by evaluating the long-term stability of PSIs in orthognathic surgery. By doing so, this review seeks to provide valuable insights into the performance of PSIs over extended periods, contributing to a more comprehensive understanding of their role in modern surgical practice.

## Materials and methods

### Study design

This systematic review was designed according to the guidelines of Preferred Reporting Items for Systematic Reviews and Meta Analyses (PRISMA) 2020. The systematic review was registered in the Open Science Framework (Registration no. 5BZT4, OSF.IO/5BZT4).

### Focused questions

This review was structured using the PICO framework. The population included patients who underwent orthognathic surgery. The intervention evaluated was the use of PSIs as the method for jaw fixation in these surgeries. Conventional fixation methods using standard plates were used as controls. Noncomparative single-arm studies were also considered for qualitative synthesis. The primary outcome assessed was the long-term skeletal stability achieved using PSIs. Secondary outcomes measured included operative time, complications and reoperations.

### Information sources and search strategy

A systematic search of online databases including Cochrane Library, Embase, Medline, PubMed, and Web of Science was performed covering the period between 26 September and 18 November 2024. The search phrase for all databases was derived from Proffit et al.^12^ Additionally, a manual search was performed in the reference list of the full text articles. Microsoft Excel was used to manage the collected data.

### Eligibility criteria

Two independent reviewers, with no competing interests, screened the titles and abstracts of the collected articles. Any disagreements or discrepancies were resolved by consensus or discussion with a third reviewer. Inclusion criteria were as follows: (1) clinical human studies with a minimum of 10 patients recruited, (2) including the use of PSIs in orthognathic surgeries, (3) reporting the brands/software the study used for designing PSIs, (4) with the presence of accuracy and long-term stability measurements and (5) a minimum of 6 months of follow-up and radiographic data.

Exclusion criteria were as follows: (1) animal or cadaveric studies; (2) case reports, technical notes, conference abstracts without full text, questionnaires and letters; (3) studies including patients with congenital craniofacial anomalies or genetic disorders or who previously had facial reconstructive procedures done (e.g. temporomandibular joint replacement, flap or cleft reconstructions); (4) studies that included patients over 70 or under 14 years of age; and (5) studies published in a language other than English.

### Data collection

The information regarding the study ID (article authors, the year and country in which the paper was published), study design, sample size, patients’ demographic data (gender, age), surgical procedure done (Le Fort osteotomy, bilateral sagittal split osteotomy), follow-up duration, brand of software used, radiographic methods used (cone beam computed tomography, lateral cephalogram, computed tomography), type and locations of PSIs, measurements of skeletal stability as the primary outcome, and secondary outcomes including duration of operation, complications and reoperations was collected.

### Quality assessment

The Risk Of Bias In Non-randomized Studies – of Interventions, Version 2 (ROBINS-I V2 2024) was used to assess the quality of 5 out of 6 studies included in this review. The quality of one other study was assessed using the Revised Cochrane Risk of Bias Tool For Randomized Trials, Version 2 (RoB 2 2019).

## Results

### Study selection and characteristics

**Fig. 1:**
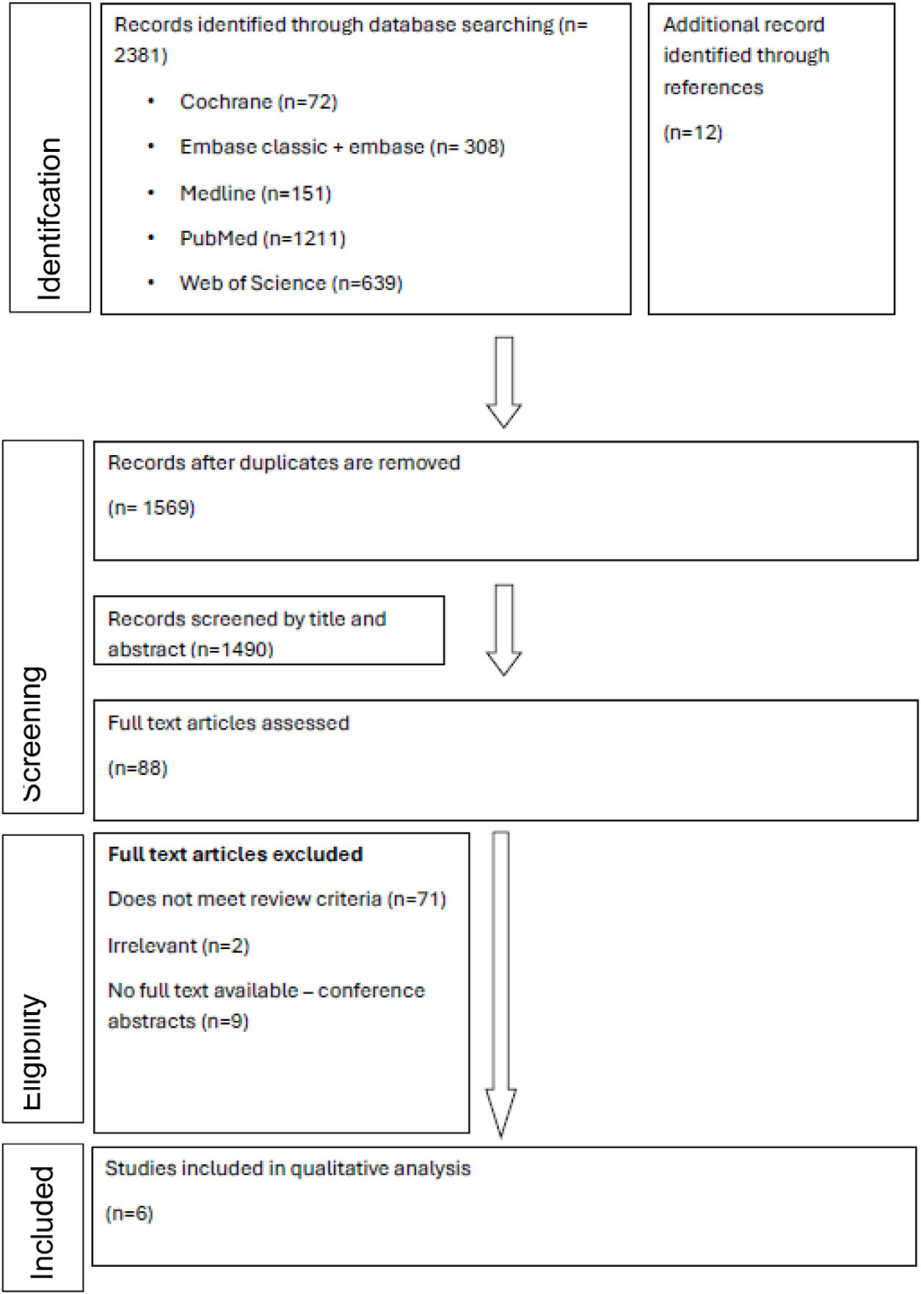
Flow chart of study search and selection.

The electronic search was conducted on 26 September 2024 and updated on 18 November 2024. The search process is summarised in Figure 1. Microsoft Excel was used to manage all search results collected from the 5 databases and to screen out duplicated studies. The total number of results retrieved from all included databases was 2,381, and the manual search of references sections of the selected studies yielded 12 additional records for a grand total of 2,393 articles. After screening for duplicates, 824 studies were removed. After screening through the titles and abstracts, another 1,481 articles were excluded. Of the remaining 88 articles, 71 were excluded because they did not meet the inclusion criteria, 2 were excluded because of their irrelevance, and 9 were excluded because they were conference abstracts. The remaining 6 studies were included in the systematic review.^13^, ^14^, ^15^, ^16^, ^17^, ^18^

### Risk of bias within included studies

**Fig. 2:**
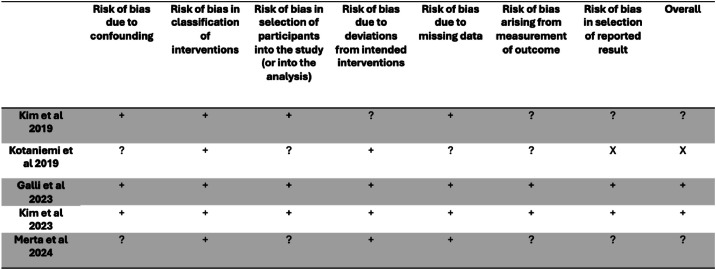
Risk-of-bias assessment (ROBINS-I V2 2024) of the included studies in the systematic review. Judgements of risk of bias are categorised into four different levels across seven domains: (+) low risk of bias; (?) moderate risk of bias; (X) serious risk of bias; (!) critical risk of bias.

**Fig. 3:**
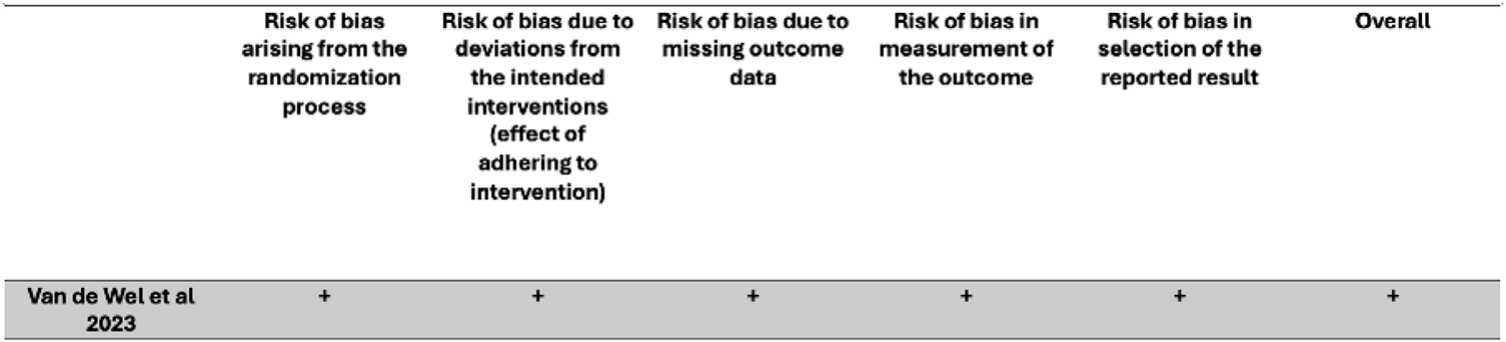
Risk-of-bias assessment (RoB 2 2019) of one of the included studies in the systematic review. Judgements of risk of bias are categorised into three different levels: (+) low risk of bias, (?) some concerns and (X) high risk of bias across five domains.

The 6 included studies were assessed for risk of bias.^13^, ^14^, ^15^, ^16^, ^17^, ^18^ Three studies^13^^,^^15^^,^^18^ were determined to have a low risk of bias, 2 studies^14^^,^^17^ were determined to have a moderate risk of bias, and 1 study^16^ was determined to have a serious risk of bias. The summaries of the risk-of-bias assessment are presented in Fig. 2, Fig. 3.

### Characteristics of eligible studies

All 6 included studies in this systematic review were published between 2019 and 2024. The studies were conducted in four countries: Finland (*n* = 2), Germany, Korea (*n* = 2), and the Netherlands (Table 1). They included a total of 171 subjects, ranging from 12 to 53 per study. The mean age of subjects across all included studies ranged from 20 to 38 years. Four studies^13^^,^^14^^,^^16^^,^^17^ were retrospective cohort studies and 2 studies^15^^,^^18^ were prospective studies, of which one^18^ was a multi-centre randomised controlled trial and another^15^ was a clinical study.

**Table 1:** Characteristics of eligible studies.

Author(s) and year published	Study design	Number of patients, gender	PSI versus control group	Mean age (range)	Software	Radiographic follow-up	PSI positions
Kim et al. 2019	Retrospective cohort	13 6M 7F	Nil (single-arm study)	22.9 (18-29)	FaceGide system	CBCT Scan T0 (2 weeks preop) T1 (3 days postop) T2 (4 months postop) T3 (1 year postop)	Maxilla Mandible
Kotaniemi et al. 2019	Retrospective cohort	51 29M 22F	Conventional miniplates (27 PSI, 24 conventional)	PSI: 29 (19-48) Conventional: 29 (19-52)	Planmeca ProModel	CT Scan (preop) Lateral cephalogram T1 (Average 4.5 months preop; 1-26 months preop) T2 (1 day postop) T3 (average 14.5 months postop; 9-34 months postop)	Maxilla
Galli et al. 2023	Retrospective cohort	15 8M 7F	Nil (single-arm study)	27.9 (16-42)	ProPlan CMF 3.0	CT Scan T0 (preop) T1 (<5 days postop) T2 (1 year postop)	Maxilla
Kim et al. 2023	Prospective clinical study	12 5M 7F	Conventional titanium plates (6 PSI, 6 conventional)	PSI: 20 (15-27) Conventional: 21 (19-23)	Ondemand CAD/CAM and Doctor Check	CBCT Scan T0 (2 weeks preop) T1 (7 days postop) T2 (6 months postop)	Maxilla Mandible
van der Wel et al. 2023	Multi-centre randomised controlled trial	27 13M 14 F	Conventional miniplates (14 PSI, 13 conventional)	PSI: 28.6 Conventional: 26.8	Maxilim v2.3	CBCT Scan T1 (preop) T2 (average 2 weeks postop; 9-16 days) T3 (average 1 year postop; 10-15 months)	Maxilla
Merta et al. 2024	Retrospective cohort	53 12M 41F	Conventional miniplates (21 PSI, 32 conventional)	PSI: 38 (25-53) Conventional: 39 (21-56)	Planmeca ProModel	Lateral cephalogram T1 (few months prior to the surgery) T2 (1 day postop) T3 (>6 months postop)	Mandible

Regarding the software used to design the PSIs, 1 study used ProPlan CMF 3.0,^13^ one study used Facegide System,^14^ 1 study used Ondemand CAD/CAM and Doctor Check,^15^ 2 studies used Planmeca ProModel,^16^^,^^17^ and 1 study used Maxilim v2.3.^18^

Regarding the collection of radiographic data, 3 studies used cone beam computed tomography (CBCT),^14^^,^^15^^,^^18^ 1 study used computed tomography (CT),^13^ and 2 studies used lateral cephalograms.^16^^,^^17^ Radiographic data on PSI long-term stability for all included studies covered a minimum of 6 months of follow-up. Four of the included studies have included such radiographic record at 1 year postop^13^^,^^14^^,^^16^^,^^18^ while the other 2 included radiographic record at 6 months postop.^15^^,^^17^

Two of the included studies had used PSIs in both the maxilla and the mandible.^14^^,^^15^ Three others had used PSIs only for the maxilla,^13^^,^^16^^,^^18^ and 1 study had used PSIs only in the mandible.^17^ The study characteristics are presented in Table 1.

### Primary outcome: long-term stability of maxillary and mandibular procedures using PSI

The primary outcome of long-term stability of fixation using PSI includes skeletal stability assessed in terms of linear and angular changes of both the maxilla and the mandible, as well as rotational and transverse changes of the maxilla (Table 2, Table 3, Table 4).

**Table 2:** Long-term stability of maxillary procedures with PSI: linear, transverse and angular changes.

				Linear movement		
Author(s) and year published	PSI location	Radiographic time frame	Landmark/ references	Absolute difference in translation (X axis / anterior-posterior) (mean, SD, *P*)	Absolute difference in translation (Y axis / cranial-caudal) (mean, SD, *P*)	Direct distance (mean, SD, *P*)
Kim et al. 2019	Maxilla	1-year follow-up CBCT scan compared with 3 days postop	Midpoint of upper central incisor roots	/	/	0.529 mm, 0.982, .352
Kotaniemi et al. 2019	Maxilla	1-year follow-up lateral cephalogram compared with 1 day postop	Upper central incisal edge	0.27 mm, 1.34, .70	1.07 mm, 0.95, .47	/
Kim et al. 2023	Maxilla	6-month follow-up CBCT scan compared with 7 days postop	Upper left central incisal edge	/	/	0.588 mm, 0.733, Not significant
van der Wel et al. 2023	Maxilla	1-year follow-up CBCT scan compared with 2 weeks postop	Most mesial point of upper central incisal edge	0.5 mm, 0.2-0.8, >.05	0.3 mm, 0.1-1.3, >.05	0.656 mm


				Transverse changes		
				Difference in arch width at anterior (palatal bone at canine level) (mean, SD, min-max, *P*)		Difference in arch width at posterior (palatal bone at first molar level) (mean, SD, min-max, *P*)
Galli et al. 2023	Maxilla	1-year follow-up CT scan compared with <5 days postop	Anterior/ posterior arch	0.05 mm, 0.48, –0.88; 1.01 (mm), .5614		0.16 mm, 0.7, –0.93; 1.22 (mm), .4543

				Angular Difference		
				Absolute difference in SNA angle (mean, SD, *P*)		
Kotaniemi et al. 2019	Maxilla	1-year follow-up lateral cephalogram compared with 1 day postop	Upper central incisal edge/SNA	0.57°, 0.74, .44

**Table 3:** Long-term stability of maxillary procedure with PSI: rotational changes.

				Rotational movement		
Author(s) and year published	PSI location	Radiographic time frame	Landmark/references	Movement of maxilla in pitch axis (median, Q1-Q3, *P*)	Movement of maxilla in roll axis (median, Q1-Q3, *P*)	Movement of maxilla in yaw axis (Median, Q1-Q3, P value)
van der Wel et al. 2023	Maxilla	1-year follow-up CBCT scan compared with 2 weeks postop	Most mesial point of upper central incisal (U1) edge and most inferior point of upper right (#16) and left molar (#26)	0.1°, 0.0-0.4, >.05	0.5°, 0.1-1.1, >.05	0.2°, 0.1-0.6, <.01

**Table 4:** Long-term stability of mandibular position with PSI: linear and angular changes.

				Linear movement
Author(s) and year published	PSI location	Radiographic time frame	Landmark/ references	Absolute direct distance (mean, SD, *P*)
Kim et al. 2023	Mandible	6-month follow-up CBCT scan compared with 7 days postop	Point B	0.729 mm, 0.514, Not significant
				**Angular Difference**
				**Absolute difference in SNB angle (mean, SD, *P*)**
Merta et al. 2024	Mandible	More than 6-month follow-up lateral cephalogram compared with 1 day postop	SNB	0.05°, 1.06, .38

#### Linear change

For the assessment of maxillary stability achieved using PSI, the absolute difference in the translational movement of the upper central incisor in the anteroposterior (x-axis) and vertical or cranial-caudal (y-axis) dimensions was reported in 2 of the included studies.^16^^,^^18^ The reported mean movement on the x-axis and the y-axis ranged, respectively, from 0.27 mm to 0.5 mm and from 0.3 mm to 1.07 mm.

Apart from the x-axis and y-axis, linear change was also studied using the direct distance difference of the selected landmarks. The direct distance difference between the pre-operative and post-operative position of the upper central incisor was reported in 3 studies,^14^^,^^15^^,^^18^ with a reported range of 0.529 mm to 0.656 mm.

For the long-term stability of PSI in the mandible, 1 study^15^ reported a mean change of 0.729 mm in the linear movement of point B between 7 days and 6 months postoperatively.

#### Angular change

One study^16^ reported the stability of PSI in the maxilla in terms of the change of the SNA (Sella-Nasion-Point A) angle, with a mean change of 0.57°. One study^17^ examined the stability of PSI in the mandible in terms of the absolute difference of the SNB (Sella-Nasion-Point B) angle, with an absolute mean difference of 0.05°.

#### Transverse change

Regarding the transverse change of the maxilla, 1 study^13^ reported the difference in the arch width of the maxilla regarding anterior width (palatal bone at the canine level) and posterior width (palatal bone at the first molar level). The reported mean change in the anterior and posterior arch widths was 0.05 mm and 0.16 mm, respectively.

#### Rotational change

One study^18^ compared the rotation of maxilla around the upper central incisor in 3 axes, specifically pitch, roll and yaw, at 2 weeks and 1 year postoperatively. The reported mean change was 0.1°, 0.5° and 0.2° for the pitch, roll and yaw, respectively.

### Secondary outcomes: duration of operation, complications and reoperations

#### Duration of the operation

One study reported the mean operation time.^15^ The mean operation time for the cases using PSI was 5.76 hours, compared with the mean of 6.83 hours for cases using conventional miniplates and screws, representing a 15.7% reduction in time per procedure. The other 5 included studies did not mention the duration of operation (Table 5).

**Table 5:** Quantitative summary with pooled weighted mean for long-term stability outcomes following orthognathic surgeries using PSI.

Outcomes	Axis/parameter	No. of studies	No. of patients (PSI)	Quantitative summary estimate (pooled weighted mean*)	Range across studies
Linear movement					
Linear translation (maxilla)	x-axis (antero-posterior)	2	65	0.38 mm*	0.27-0.50 mm
	y-axis (cranial-caudal)	2	65	0.69 mm*	0.30-1.07 mm
Direct linear distance (maxilla)	U1 reference point	3	53	0.59 mm*	0.53-0.66 mm
Linear translation (mandible)	Point B	1	12	0.73 mm	-
Transverse movement					
Transverse change (maxilla)	Anterior arch width (canine level)	1	15	0.05 mm	−0.88 to 1.01 mm⁎⁎
	Posterior arch width (molar level)	1	15	0.16 mm	−0.93 to 1.22 mm⁎⁎
Angular movement					
Angular change (maxilla)	SNA	1	51	0.57°	-
Angular change (mandible)	SNB	1	53	0.05°	-
Rotational movement					
Rotational change (maxilla)	Pitch	1	27	0.10°	0.0-0.4°
	Roll	1	27	0.50°	0.1-1.1°
	Yaw	1	27	0.20°	0.1-0.6°

⁎ Pooled weighted means calculated only where 2 or more studies reported comparable parameters.

⁎⁎ The range reported min-max values within the study rather than cross-study variations.

#### Complications and reoperations

Three studies reported no complications and requiring no reoperations in their cumulative cohort of 46 subjects.^14^, ^15^, ^16^ The other 3 studies did not provide any information regarding complications or the need for reoperations^13^^,^^17^^,^^18^ (Table 6).

**Table 6:** Complications and reoperations of PSI for orthognathic surgery.

Author(s) and year published	PSI positions	Mean operation time (mean + SD)	Complications	Reoperations
Kim et al. 2019	Maxilla Mandible	Not reported	No	No
Kotaniemi et al. 2019	Maxilla	Not reported	No	No
Galli et al. 2023	Maxilla	Not reported	Not reported	Not reported
Kim et al. 2023	Maxilla Mandible	PSI: 5.76 ± 0.43 h Conventional miniplates: 6.83 ± 0.72 h	No	No
van der Wel et al. 2023	Maxilla	Not reported	Not reported	Not reported
Merta et al. 2024	Mandible	Not reported	Not reported	Not reported

## Discussion

The key findings of this systematic review were that (1) the discrepancy in bone movements in orthognathic surgery using PSIs was less than 2 mm in linear and transverse movements and within 1° in rotational movements and angular differences; (2) the mean duration of orthognathic surgical procedures using PSIs was, on average, 15.7% shorter than for operations using conventional miniplates (albeit based on only 1 comparative study that reported operation time); and (3) no significant complications and or need for reoperation after orthognathic surgical procedures using PSIs.

In the modern era of digital dentistry, CAD/CAM has evolved into an essential and pivotal role in aiding numerous dental procedures such as prosthodontic and orthodontic planning and treatments. Through the combined efforts of oral maxillofacial surgeons and biomechanical engineers, CAD/CAM manufactured personalised surgical cutting guides, and PSIs have been used in orthognathic surgeries to position patients’ jaws in the planned positions for the past 10 years, replacing the traditional method of using conventional pre-bent miniplates and splints.^19^ Numerous studies have analysed and confirmed the accuracy of the use of PSIs in orthognathic surgeries, such as Heufelder^4^ reporting a deviation between preoperative and postoperative maxilla positions of just 0.39 mm (median). It is worth noting, however, that most of the available literature determined the accuracy of PSIs by comparing very short-term postoperative results with preoperative data. Thus, there is a lack of consensus on the long-term stability of PSIs and hence uncertainties about the long-term outcome. This systematic review attempted to investigate and objectively summarise the long-term stability of PSIs.

Achieving long-term stability while fixating the jaw in the planned position for an extended period of time is one of the major measures of success in orthognathic surgeries. A clinically acceptable translational threshold of <2 mm has been widely reported and accepted in orthognathic surgery literature.^12^ Rotational changes of >4° have been considered to be significant in terms of postoperative skeletal instability.^20^^,^^21^, ^22^, ^23^, ^24^, ^25^ We theorise that with the assistance of better preoperative preparation and less complex intraoperative procedures, PSIs yield similar, if not better, long-term outcomes when compared with traditional miniplate fixation.

Miniplate fixation has been the go-to option for jaw fixation in orthognathic surgeries for a very long time, and the literature has documented its successful long-term stability in orthognathic jaw repositions.^26^, ^27^, ^28^ Gaitan-Romero^29^ evaluated the long-term stability of miniplates with a minimum of 5 years of review time, showing the mean sagittal relapse of Point A ranged from –0.1 mm to –0.94 mm and the mean vertical relapse of Point A ranged from 0.37 mm to –3mm. The study also has shown that the mean changes of angle SNA ranged from –1.63° to 0.75°. Comparing these results with the results in this review, it is notable that the long-term stability of PSIs is similar to, if not better than, traditional miniplate fixation.

The promising results in the long-term stability of PSIs can be accredited to numerous factors. The conventional miniplate fixation method has limitations in multiple steps of presurgical planning. The use of sagittal lateral cephalograms in presurgical planning raises the possibility of errors because the assessment of the jaws is done in only a 2-dimensional (2D) manner, and skeletal anatomy and facial structure in the roll axis or facial asymmetry are left unassessed. Furthermore, true clinical canting and horizontal reference may not be accurately captured by physical facebow records, leading to inaccurate relationships between the jaws and the cranium and subsequently imprecisions in the mounted records of casts. Given that the repositioning of the jaws relies on surgical wafers, inaccurate mounting may lead to incorrect realignment of the jaw and consequent mistakes in the opposing jaw repositioning in bimaxillary surgery.^30^

Advancements in technology have allowed improvement and refinement in diagnosis and preoperative surgical planning. The inclusion of novel CBCT and CAD/CAM technologies has opened the horizons to unlimited possibilities. The ability to plan and design in 3 dimensions proved to be groundbreaking and created an opportunity to move away from the limitations of the conventional approach, bypassing 2D cephalograms, physical facebows and cast mounting.^31^ The digitalisation also allowed surgeons and engineers to use 3-dimensional (3D) virtually constructed models to plan bony movements with computerised simulation of intraoperative surgical steps. Inside the operating room, laboratory-fabricated wafers may be error-prone because the repositioning of the jaws relies on the planned jaw relationships. Replacing stonecast laboratory-fabricated wafers with fine and accurate 3D-printed or milled surgical guides and PSIs avoids the reliance on opposing occlusion and the influence of stability of the condyle-fossa relationship,^30^^,^^32^ with existing studies having shown them to be superior in terms of accuracy.^33^^,^^34^

Information gleaned from this data analysis may serve as a catalyst in persuading more oral maxillofacial surgeons to explore the use of PSIs in their practice and be a propagating factor in a paradigm shift in the industry regarding the primary method selection when it comes to orthognathic surgery.

Duration of operation is another fundamental objective in assessing PSIs’ surgical outcomes. One analysed study noted the mean duration of operation when using PSIs to be almost 16% less than that of the same procedure using conventional miniplates.^15^ It is notable, however, that the other 5 studies did not report data on operation time, and hence the aforementioned result should be interpreted with caution. Still, this result does align with multiple studies as described by Heufelder^4^ indicating a reduction in surgery time by approximately 30-45 minutes and Sanchez-Jauregui^35^ reporting a 36.5-minute reduction in mean operating time.

The reduced surgery time when using PSIs can be attributed to numerous factors. First, because PSIs are prepared before the operation, bone plate bending is no longer required to be performed during surgery. The presence of CAD/CAM-prepared surgery guides provides indications for the location of the screws, requiring surgeons to drill all the screw holes at once and to tighten the screws when the PSIs are well positioned. Using PSIs also eliminates the need for intermaxillary fixation during the procedure, which requires time-consuming wire manipulation.

Despite the reduction in intraoperative time, it is worth noting that the process of designing and fabricating PSIs beforehand is itself time consuming. To fabricate patient-specific surgical guides and implants, multiple steps must be approved and done before the final product is delivered to the hospital, such as discussing and formulating the designs with biomechanical engineers and designing the guides and plates using the software. Li has reported 30-60 minutes were required to plan the surgery using computer-aided surgical simulation and an additional 70-95 minutes to design the personal orthognathic surgical guide system.^21^ Likewise, Hanafy has reported the average planning time for the computer-aided surgery required 113 minutes.^36^

Producing surgical guides and plates through 3D printing or milling also takes time, and additional delays may occur if the materials need to be transported to the hospital instead of being produced in-house. Espino-Segura-Illa has reported a minimum of 5 working days was required for the printing and delivery of the patient-specific guides and plates to the place of operation.^3^ Similarly, Leung has reported that the preparation turnaround time spans from 4 to 6 weeks, also noting that trained surgeons required 3 to 4 hours to conduct VSP and to design surgical guides and PSIs.^8^ Therefore, consideration should be given to the time required for planning and delivering the PSIs before the surgical operation despite having the benefit of a reduction in intraoperative time.

Complications and reoperation rates in the long term are pivotal parameters in the determination of treatment success for orthognathic surgeries. The studies included in this review have indicated there is a minimal chance of complications and the need for reoperation.

CBCT and CT provide a 3D visualisation of the bony anatomy and quality of bone, and VSP and CAD/CAM allow a more accurate and precise preoperative planning. This subsequently translates into accurate intraoperative jaw positioning and fixation. Using conventional miniplates requires on-the-table adjustments, including removing more bony structure and technique-sensitive miniplate bending. Furthermore, conventional miniplate fixation primarily depends on manually positioning and securing the segments. To deliver an aesthetically pleasing result, it involves intraoperative estimation and depends heavily on the surgeon’s judgement.^37^ This may contribute to a significant deviation from the planned surgical movements, leading to malocclusion and the need for reoperation. Comparing the rates of complications and instances of reoperation for the procedures using conventional miniplates reported in the extant literature with the same as reported in the studies included in this analysis indicates an advantage provided by using PSIs in these terms.

It is worth mentioning that one of the included studies^15^ mentioned a control group of patients operated on using conventional miniplates and reported complication for 1 patient in that group. In this patient, exposure of the right mandibular plate and inflammation of the surrounding tissue were observed postoperatively at 8 weeks. Surgical debridement and reoperational changes to the conventional plate were performed, and normal healing was observed at the 6-month follow up. Another study^16^ also included such a control group and reported the need for reoperation in 2 patients from the miniplate group: open bite was noticed immediately in one patient and unsatisfactory bite was observed immediately postoperatively in the other patient. These observations align with other studies. For instance, in the study by Suojanen et al.,^38^ none of the 31 maxillae positioned using PSIs required immediate reoperation because of malocclusion, whereas 3 out of 37 miniplate-positioned maxillae required immediate reoperation (though the difference was not statistically significant).

This systematic review provides insight into the long-term stability of the use of PSIs in orthognathic surgery. The limitations of this study must be noted, however.

First, despite a significant number of studies analysing the accuracy of PSIs, there is a lack of existing literature that analyses the stability of PSIs in the long run, with a minimum of 6 to 12 months of follow-up including radiographic data comparing preoperative and postoperative conditions. It is recommended that future studies plan to follow up their patients who underwent orthognathic surgery with the use of PSIs for at least 6 to 12 months to evaluate the long-term success and stability of PSIs.

Second, some of the included studies used 2D radiographic data such as lateral cephalograms while others used 3D radiographic data such as CBCT. Three-dimensional radiographic methods such as CBCT and CT provide a more comprehensive, complex and complete analysis of surgical movements and outcomes. Having the aid of 3D radiographic data is fundamental and pivotal in evaluating the accuracy and stability of PSIs. Hence it is recommended that, if possible, future studies aim to include the use of 3D radiographic methods.

Lastly, the bulk of the studies examined in this systematic review consisted of cohort studies and are retrospective in nature, hence they may encounter certain limitations when attempting to establish causality and account for potential confounding variables. As a result, it is important to approach the findings with a critical and nuanced perspective. To gather more definitive evidence, future studies with a prospective design and randomised clinical trials should be carried out to evaluate precisely the long-term success and stability of PSIs. Furthermore, the small sample sizes in some of the included studies may compromise their statistical power, potentially resulting in a failure to identify significant outcomes.

## Conclusion

To achieve a successful long-term orthognathic surgery outcome, aside from the primary method of jaw fixation, other aspects and factors, including the amount of maxillary of mandibular movements, may need to be considered. This systematic review highlights the long-term stability of the use of PSIs in orthognathic surgeries. Because of the heterogeneity in methodology and lack of studies with a follow-up duration of more than 24 months, a definite conclusion on the long-term stability of PSI could not be sufficiently derived. However, a qualitative synthesis of the current systematic review suggests that the use of PSIs has a clinically acceptable level of stability in the medium-to-long term and has proved to be a viable method of fixation in orthognathic surgeries. While PSI shows possibly better surgical outcomes, more randomised clinical trials on the stability of PSI, with a longer follow-up period, are required.

## Author contributions

*Conceptualisation:* All authors.

*Design:* All authors.

*Material preparation:* All authors.

*Data collection:* All authors.

*Data analysis:* All authors.

*Writing—initial draft:* Wu.

*Writing—review and editing:* All authors.

## Funding

This research did not receive any specific grant from funding agencies in the public, commercial, or not-for-profit sectors.

## Conflict of interest

None disclosed.
